# Real‐World Evaluation of Outcomes and Safety of Elexacaftor/Tezacaftor/Ivacaftor in Pediatric Patients With Cystic Fibrosis: A Retrospective Study

**DOI:** 10.1111/cts.70373

**Published:** 2025-10-03

**Authors:** Nicola Perrotta, Luigi Angelo Fiorito, Rossella Gentile, Roberta Vescovo, Alfonso Piciocchi, Patrizia Troiani, Roberto Poscia, Giuseppe Cimino

**Affiliations:** ^1^ Department of Physiology and Pharmacology “V. Erspamer” Sapienza University Rome Italy; ^2^ Pharmacy Unit AOU Policlinico Umberto I – Sapienza University Rome Italy; ^3^ Department of Chemistry and Technology of Drugs Sapienza University Rome Italy; ^4^ Biostatistics Unit GIMEMA Foundation Rome Italy; ^5^ Regional Cystic Fibrosis Center AOU Policlinico Umberto I – Sapienza University Rome Italy; ^6^ Clinical Research Unit AOU Policlinico Umberto I – Sapienza University Rome Italy; ^7^ Interdepartmental Center for Rare Diseases AOU Policlinico Umberto I – Sapienza University Rome Italy

**Keywords:** cystic fibrosis, effectiveness, elexacaftor‐tezacaftor‐ivacaftor, pediatric setting, safety

## Abstract

Elexacaftor/Tezacaftor/Ivacaftor (ETI) therapy has significantly improved clinical outcomes in people with cystic fibrosis (PwCF) carrying at least one Phe508del CFTR mutation. However, real‐world evidence on the safety and effectiveness of ETI in pediatric populations remains limited, particularly in children with more severe disease phenotypes who are often excluded from clinical trials. We analyzed clinical, functional, and microbiological data from pediatric CF patients aged 6–17 years treated with ETI between October 2022 and March 2024. Lung function (ppFEV_1_, ppFVC), nutritional status (BMI, BMI *z*‐score), sweat chloride concentration (SwCl), quality of life (CFQ‐R), pulmonary exacerbations (PEx), and airway pathogens were assessed at baseline and after 6–12 months. Adverse events (AEs) and therapy discontinuations were also recorded. Twenty‐four patients (*n* = 10 aged 6–11 years; *n* = 14 aged 12–17 years) were included. At 12 months, mean ppFEV_1_ increased by 15% (*p* = 0.013), BMI by 2.4 kg/m^2^ (*p* = 0.16), BMI weight z‐score by 0.33 (*p* = 0.63), and height *z*‐score by −0.33 (*p* = 0.72), SwCl decreased by 46 mmol/L (*p* < 0.001), and CFQ‐R respiratory domain improved by 14 points (*p* < 0.001). PEx rates decreased by 27.6% after 12 months. Reductions in airway pathogen prevalence, particularly *Staphylococcus aureus* and *Pseudomonas aeruginosa* , were observed. AEs occurred in 14.8% (*n* = 4) of patients, were mild‐to‐moderate, and resolved with dose reduction. ETI therapy was associated with marked improvements in lung function, nutritional status, and quality of life, along with reductions in PEx and airway pathogens, in a real‐world pediatric CF cohort. These findings support ETI use in this population, with careful AE monitoring and dose adjustment when needed.

**Study Highlights**

- What is the current knowledge on the topic?
  ○ Elexacaftor/tezacaftor/ivacaftor (ETI) is a transformative triple‐combination CFTR modulator that has significantly improved outcomes in people with cystic fibrosis (PwCF) carrying at least one F508del allele. Phase III trials demonstrated robust benefits in pulmonary function, nutritional status, and quality of life in both adults and children. However, pediatric real‐world evidence (RWE) remains limited, particularly in European cohorts, and there is a paucity of knowledge regarding the impact of ETI on microbiological outcomes, growth trajectories, and long‐term safety when therapy is initiated during critical developmental stages.
- What question did this study address?
  ○ This study assessed the real‐world effectiveness and safety of ETI over 12 months in Italian pediatric PwCF aged 6–17 years, stratified by age group and baseline lung function. It examined clinical, functional, microbiological, and patient‐reported outcomes, while also exploring the feasibility of dose adjustments in managing adverse events without compromising therapeutic benefit.
- What does this study add to our knowledge?
  ○ ETI was associated with rapid and sustained improvements in lung function (+15% ppFEV1 at 12 months), sweat chloride (−44 mmol/L), and quality of life (+14 CFQ‐R points), with a 27.6% reduction in pulmonary exacerbations and significant decreases in *Pseudomonas aeruginosa* and *Staphylococcus aureus* colonization. BMI improved progressively, though growth z‐scores showed only modest changes, underscoring the multifactorial nature of CF‐related growth impairment, possibly linked to CFTR dysfunction in the GH–IGF‐1 axis. Adverse events were infrequent, mild‐to‐moderate, and largely resolved with dose reduction, which preserved efficacy.
- How might this change clinical pharmacology or translational science?
  ○ These findings provide strong real‐world support for ETI use in pediatric CF, reinforcing its role as standard of care within routine European practice. The study highlights microbiological and quality‐of‐life benefits often underreported in trials, and emphasizes that dose tailoring in response to adverse events can maintain long‐term effectiveness. Importantly, early ETI initiation during growth and lung development may confer durable benefits, warranting further translational research into its impact on growth‐endocrine pathways and long‐term safety in children.

## 1 Introduction

Cystic fibrosis (CF) is a rare autosomal recessive disorder caused by mutations in the cystic fibrosis transmembrane conductance regulator (CFTR) gene, leading to defective chloride and bicarbonate transport across the apical membrane of epithelial cells [1]. This dysfunction results in the production of thick, viscous secretions that affect multiple organs, especially the lungs and pancreas, and promote chronic bacterial infections and inflammation [2, 3].

Until the recent advent of CFTR modulators, CF treatment focused predominantly on symptom management. The triple‐combination therapy of elexacaftor, tezacaftor, and ivacaftor (ETI) represents a significant advancement in precision medicine for CF, offering direct modulation of CFTR protein function. Since its approval by the Italian Medicines Agency (AIFA) in 2021, ETI has been widely used in PwCF with at least one Phe508del allele [4].

Phase III clinical trials have demonstrated substantial improvements in pulmonary function and nutritional outcomes with ETI in both adults and children [5, 6, 7, 8]. Consequently, in September 2022, AIFA approved the use of ETI in children aged six and older who have at least one copy of the Phe508del mutation [9].

Nevertheless, further real‐world data are required to facilitate a more comprehensive understanding of its impact in everyday clinical practice, particularly in pediatric populations where treatment initiation often coincides with critical stages of growth and lung development.

Recent observational studies and reviews have begun to address this gap [10, 11]. However, published cohorts remain limited in size and geographic scope. These studies underscore the substantial benefits observed, while simultaneously highlighting considerable variability in individual responses within real‐life settings. The study emphasizes a need for further data to be collected from a range of healthcare systems and patient populations.

The present study aims to contribute to this growing body of evidence by providing a single‐center, retrospective evaluation of ETI outcomes and safety in Italian pediatric patients with CF, stratified by age and baseline characteristics, and followed for 12 months. This methodological approach permits the comparison of our findings with those of RCTs as well as with prior real‐world studies, thus offering a clinically relevant perspective on the role of ETI in routine care.

## 2 Material and Methods

### 2.1 Study Design and Objectives

This study is a retrospective, single‐center, observational cohort investigation conducted at the CF Regional Reference Center, Umberto I University Hospital, Rome, Italy, between October 2022 and March 2024. It aimed to evaluate the effects of ETI therapy in pediatric patients with CF aged 6–17 years who were followed for the entire duration of the study. Patients were stratified by age (6–11 and 12–17 years) and by baseline ppFEV_1_ (< 40%, 40%–70%, > 70%).

ETI treatment was administered to all eligible patients according to national guidelines based on body weight. Children weighing less than 30 kg received Elexacaftor 100 mg once daily, Tezacaftor 50 mg once daily, and Ivacaftor 75 mg twice daily. For those weighing 30 kg or more, the doses were increased to Elexacaftor 200 mg once daily, Tezacaftor 100 mg once daily, and Ivacaftor 150 mg twice daily.

The primary focus of the study was to assess the absolute changes in lung function in accordance with the international standards [12], specifically the percent predicted forced expiratory volume in 1 s (ppFEV_1_) and percent predicted forced vital capacity (ppFVC), measured at 6‐ and 12‐month post‐therapy initiation. The study also examined pulmonary exacerbation rates (PEx), defined as hospitalizations or the need for intravenous antibiotics due to worsening respiratory symptoms. PEx data were collected for 12 months before and after starting treatment. Baseline lung function was obtained from the last spirometric measurement taken before the start of therapy. This study was conducted at a single tertiary pediatric CF center in Italy, selected to ensure uniformity in clinical management, data collection, and follow‐up protocols. While this design minimized variability, it also limited the sample size and the generalizability of the findings.

Secondary outcomes included changes in nutritional status [body mass index (BMI), weight z‐score and height *z*‐score], sweat chloride levels (SwCl) measured by quantitative pilocarpine iontophoresis test (QPIT), and quality of life as assessed through the Cystic Fibrosis Questionnaire‐Revised (CFQ‐R), administered at baseline, 6 months, and 12 months. Data on adverse events, microbial colonization, mortality, and the rate of lung transplantation were also collected as secondary endpoints.

### 2.2 Participants

The inclusion criteria for participants were specific to subjects aged 6–17 years with confirmed CF and homozygosity or heterozygosity for the Phe508del CFTR mutation. Exclusion criteria included age < 6 years, need for mechanical ventilation, severe hepatic dysfunction, or history of organ transplantation. Clinical and analytical data such as lung function tests (ppFEV_1_, ppFVC), alanine aminotransferase (ALT), aspartate aminotransferase (AST), creatine phosphokinase, and bilirubin levels were collected from electronic medical records, along with details on the CFTR mutation genotype, treatment regimens, and complications related to CF, such as bronchial bacterial colonization and hospitalizations.

The study was approved by the local Ethics Committee (protocol number 7096/CE) and conducted in accordance with the Declaration of Helsinki of 1975 (as revised in 1983). Written informed consent was obtained from the parents or legal guardians of each participant, and the children were asked to provide assent to participate according to national legislation.

### 2.3 Statistical Analysis

Descriptive statistics were used to summarize patient characteristics. Continuous variables were analyzed using ANOVA and Shapiro–Wilk tests for normality. To support the analysis, scatter plots, boxplots, and dumbbell plots were performed for each variable mentioned, allowing for a visual assessment of the relationships and distributions. All tests were 2‐sided, accepting *p* < 0.05 as statistically significant, and confidence intervals were calculated at the 95% level. The manuscript content was created by the authors. AI was used to improve figures' readability. All analyses were performed using R software (R Core Team 2022) [13].

## 3 Results

Twenty‐seven pediatric PwCF (median age 12.0 years; 66.6% female) were included. Most (88.9%) were homozygous for the Phe508del mutation, and 81.5% were ETI‐naïve. Baseline pancreatic insufficiency was present in 100% of patients. Patients were divided into two age groups: 6–11 years (*n* = 10) and 12–17 years (*n* = 17). The baseline characteristics of the study population are presented in Table 1.

**TABLE 1:** Values are mean (interquartile range) or proportion of patients as a percentage.

Demographic and clinical characteristics of the patients at baseline			
Characteristic	All patients (*n* = 27)	6–11 years (*n* = 10)	12–17 years (*n* = 17)
Female sex—*n* (%)	18 (66.6)	8 (29.6)	10 (37)
Age at baseline, mean, years	12.0 (11.0, 16.0)	9.5 (8.2, 11.0)	15.0 (14.0, 16.0)
ppFEV_1_, mean	88 (76, 102)	89 (75, 106)	85 (78, 101)
ppFEV_1_ category, *n* (%)			
< 40%	1 (3.7%)	—	1 (5.9%)
40 to < 70%	4 (14.8%)	—	4 (23.5%)
> 70%	22 (81.5%)	10 (100%)	13 (48.1%)
ppFVC, mean	109 (91, 118)	115 (109, 118)	103 (87, 114)
BMI, mean, kg/m^2^	18.4 (16.0, 21.2)	17.3 (14.6, 19.2)	20.1 (17.2, 22.7)
Weight‐for‐age *z*‐score, mean	0.05 (−0.54, 0.80)	0.13 (−0.56, 1.05)	0.05 (−0.49, 0.53)
Height‐for‐age *z*‐score, mean	−0.24 (−0.71, 0.37)	−0.61 (−0.72, −0.09)	0.08 (−0.68, 0.66)
SwCL, mean, mmol/L	84 (53, 105)	81 (78, 88)	90 (75, 95)
CFQ‐R, mean, points	76 (68, 85)	77 (70, 85)	75 (68, 80)
Genotype—*n* (%)			
Homozygous Phe508del	24 (88.9%)	10 (100%)	14 (82.3%)
Heterozygous Phe508del	3 (11.1%)	—	3 (17.7%)
CFTR modulator history—*n* (%)			
Naïve	22 (81.5%)	9 (90%)	13 (76.5%)
Lumacaftor + Ivacaftor	5 (18.5%)	1 (10%)	4 (23.5%)
Ivacaftor	—	—	—
Tezacaftor + Ivacaftor	—	—	—

Abbreviations: BMI, body mass index; CFTR, CFQ‐R respiratory domain score, cystic fibrosis transmembrane conductance regulator; ppFEV_1_, percent predicted forced expiratory volume in 1 s; ppFVC, percent predicted forced vital capacity; SwCl, sweat chloride concentration.

### 3.1 Pulmonary Function

ETI demonstrated a rapid and sustained increase in absolute change in ppFEV_1_ of 11 percentage points at 6 months and 15% at 12 months follow‐up compared to baseline (*p* = 0.013) (Table 2). There was no statistically significant difference between the age groups at 6 months (*p* = 0.96) and at subsequent follow‐up (*p* = 0.73) (Table 3). Both patient cohorts started from similar baseline ppFEV_1_ values (87 ± 26 for children and 89 ± 17 for adolescents) and reached the same value at T6 (ppFEV_1_ = 99). A further increase was observed at T12, with final absolute changes from baseline of 18% and 14% for the 6–11‐ and 12–17‐year groups, respectively. Only one patient had a severe lung impairment (ppFEV_1_ = 30) at baseline. This value has increased by 15% and 19% after 6 and 12 months, respectively.

**TABLE 2:** Differences between measurements at baseline and measurements at 6 month and 12 month follow‐up.

Absolute change from baseline in primary and secondary endpoints				
Endpoints	T0	T6	T12	*p* ^a^
ppFEV_1_, mean (SD)	88 (21)	99 (18)	103 (13)	**0.013**
ppFVC, mean (SD)	108 (21)	116 (14)	114 (15)	0.30
BMI, mean (SD)	19.5 (4.4)	20.3 (4.6)	21.9 (4.0)	0.16
Weight *Z*‐score, mean (SD)	0.09 (0.99)	0.28 (0.96)	0.33 (0.91)	0.63
Height *Z*‐score, mean (SD)	−0.14 (1.01)	−0.32 (0.90)	−0.33 (0.87)	0.72
SwCl, mean (SD)	84 (14)	40 (17)	46 (25)	**< 0.001**
CFQ‐R, mean (SD)	76 (4)	90 (2)	90 (2)	**< 0.001**

*Note:* Significant comparisons (*p* < 0.05) are marked in bold.

Abbreviations: BMI, body mass index; CFTR, CFQ‐R Respiratory Domain Score, cystic fibrosis transmembrane conductance regulator; ppFEV_1_, percent predicted forced expiratory volume in 1 s; ppFVC, percent predicted forced vital capacity; SD, standard deviation; SwCl, sweat chloride concentration.

^a^ One‐way ANOVA.

**TABLE 3:** Absolute change in ppFEV_1_ from baseline at each study visit according to ppFEV_1_ severity (a) and age groups (b).

Differences in ppFEV_1_ by baseline severity and age at 6‐ and 12‐month follow‐up								
	ppFEV_1_ < 40	40 < ppFEV_1_ < 70	ppFEV_1_ > 70	*p* ^a^		6‐11 years	12–17 years	*p* ^a^
(a)	*n* = 1	*n* = 4	*n* = 22		(b)	*n* = 10	*n* = 17	
ppFEV_1_ T0, mean (SD)	30 (30)	64 (1)	94 (15)	**< 0.001**		87 (26)	89 (17)	0.83
ppFEV_1_ T6, mean (SD)	45 (45)	99 (3)	101 (16)	**0.005**		99 (25)	99 (14)	0.96
ppFEV_1_ T12, mean (SD)	49 (49)	110 (3)	102 (14)	0.39		105 (14)	103 (14)	0.73

*Note:* Significant comparisons (*p* < 0.05) are marked in bold.

Abbreviation: ppFEV_1_, percent predicted forced expiratory volume in 1 s.

^a^ One‐way ANOVA.

Furthermore, PwCF with baseline ppFEV_1_ between 40 and 70 showed a marked and progressive increase in ppFEV_1_ of 35% (99 ± 3) and 46% (110 ± 3) at 6 and 12 months. The absolute change in this cohort of patients was significantly higher than in patients with a baseline ppFEV_1_ > 70 (*p* = 0.005) (Table 3). PwCF with ppFEV_1_ > 70 exhibited a mean increase of 7% at 6 months, with an 8% increase observed at 12 months with respect to the baseline. Although the absolute change in mean ppFVC wasn't statistically significant (*p* = 0.30), an increase of 8% was observed after 6 months of ETI compared to baseline, with values stabilizing at 114 ± 15 after 12 months (Table 2).

### 3.2 Pulmonary Exacerbation Rate

In the 12 months prior to ETI treatment, 46.1% (*n* = 13) of patients required hospitalization or intravenous antibiotics, including 5 patients with at least one episode, 3 with at least two episodes, 2 with at least three episodes, and 2 with at least five episodes. However, 12 months after the initiation of ETI therapy, the frequency of infectious exacerbations had significantly decreased to 18.5% of PwCF (*n* = 5), with four patients experiencing more than two episodes per year 12 months after initiating ETI treatment.

### 3.3 Sweat Chloride Concentration Test

Total sweat chloride concentrations decreased rapidly through week 24, with a mean treatment difference of −44 mmol/L compared to the mean baseline value (*p* < 0.001) (Table 2).

This statistically significant improvement was maintained at 12 months after starting treatment, with values fluctuating up to 46 (±25) mmol/L. Mean values dropped below the diagnostic threshold (< 60 mmol/L) in both age groups. However, no statistically significant differences were observed among age groups (*p* = 0.87).

### 3.4 Microbial Colonization

**FIGURE 1:**
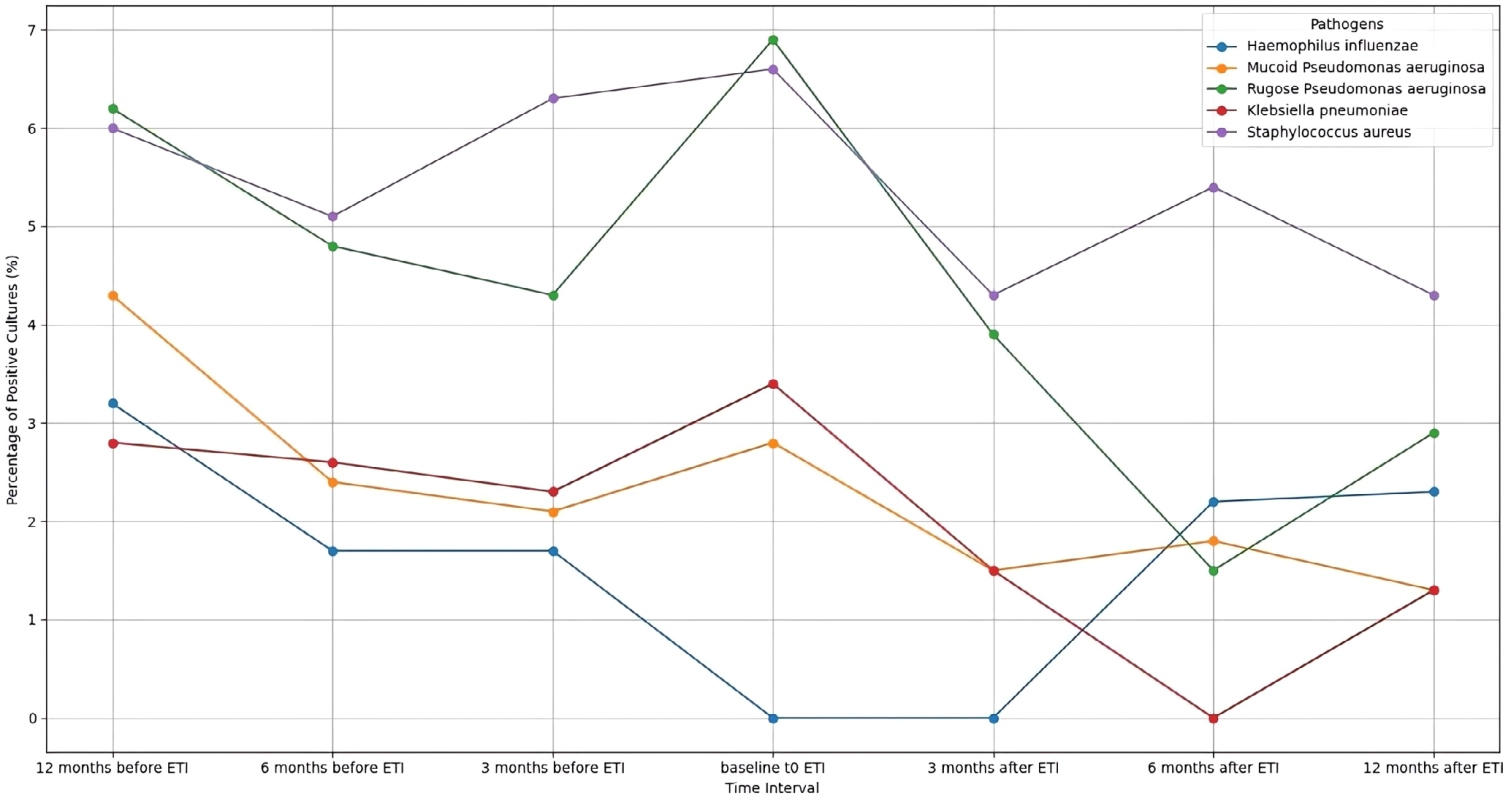
Prevalence of the most common chronic infections in pediatric CF patients (6–17 years). The line chart illustrates the percentage of colonization by specific pathogens (culture positivity for *H. influenzae* , *S. aureus* , mucoid and rugose *P. aeruginosa* , and *K. pneumoniae* ) over various time intervals relative to ETI. The x‐axis represents the time intervals, ranging from 12 months before ETI to 12 months after ETI, while the y‐axis indicates the percentage of positive bacterial cultures. The chart tracks colonization trends for *H. influenzae* , mucoid and rugose forms of *P. aeruginosa* , *K. pneumoniae* , and *S. aureus* . Distinct patterns of colonization are observed, with a general decline in percentages following ETI.

**FIGURE 2:**
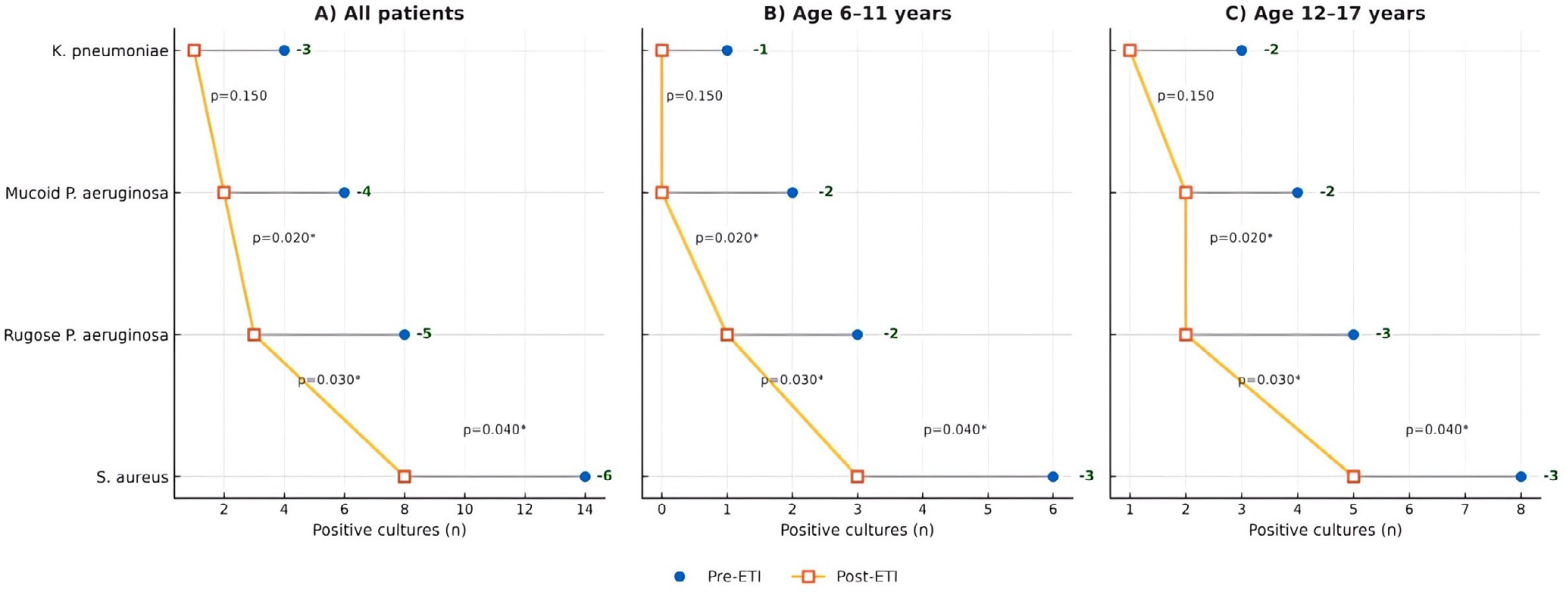
Pathogen culture positivity pre‐ and post‐ETI by age group. Tripanel dumbbell plots show the number of positive respiratory cultures for each pathogen before initiation of elexacaftor/tezacaftor/ivacaftor (ETI; blue circles) and after treatment (red open squares). Panel A: all patients (*n* = 27); Panel B: age 6–11 years (*n* = 10); Panel C: age 12–17 years (*n* = 17). Horizontal lines connect paired pre–post values; numbers on the right indicate absolute changes (Δ). Two‐sided *p* values are shown adjacent to the connectors (*p < 0.05). Significant reductions are observed for *Staphylococcus aureus* , rugose and mucoid *Pseudomonas aeruginosa* , and *Klebsiella pneumoniae* .

The prevalence of the most common chronic infections among pediatric patients was reported in Figure 1 (*n* = 27). The figure illustrates the percentage of positive bacterial cultures from 12 months prior to treatment to 12 months post‐ETI, with a three‐month interval between each follow‐up. All the pathogens analyzed demonstrated a significant reduction in the number of infections following the initiation of ETI treatment, particularly after 6 months of treatment. The percentage of infections decreased 12 months after ETI, from 6.6% to 4.3% for *S. aureus* , from 6.9% to 2.9% for *rugose P. aeruginosa*, and from 3.4% to 1.3% for *K. pneumoniae* (Figure 1). Tripanel dumbbell plots showed the number of positive respiratory cultures for each pathogen prior to the initiation of ETI and following treatment, stratified according to age groups (Figure 2).

### 3.5 Nutritional Status

**FIGURE 3:**
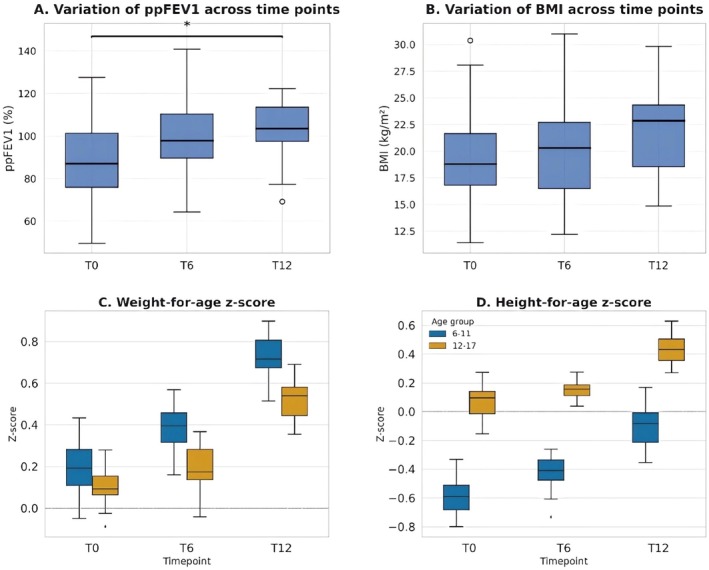
Longitudinal variation of clinical and auxological parameters. Panels show (A) variation of ppFEV_1_ across time points, (B) variation of BMI across time points, (C) variation of weight‐for‐age *z*‐score by age group (6–11 years and 12–17 years), and (D) variation of height‐for‐age z‐score by age group (6–11 years and 12–17 years). Data are presented as box plots (median, interquartile range, and overall range). Assessments were performed at baseline (T0), 6 months (T6), and 12 months (T12). A significant improvement was observed for ppFEV_1_ (panel A), with *p* < 0.05 for the comparison between T0 and T12. No statistically significant differences were detected for BMI, weight‐for‐age *z*‐score, or height‐for‐age *z*‐score (panels B–D).

Although ETI therapy resulted in a progressive improvement in BMI (+0.8 kg/m^2^ and +2.4 kg/m^2^ at 6 and 12 months, respectively), the absolute change in total BMI (*p* = 0.16), weight *z*‐score (*p* = 0.63), and height *z*‐score (*p* = 0.72) was not statistically significant (Table 2). The modest *z*‐score increase may reflect the chronicity of growth delay in CF. The distribution of the *z*‐score for weight and height from baseline at each study visit, stratified by age group, is shown in Figure 3.

### 3.6 CFQ‐R Respiratory Domain Scores

The CFQ‐R scores demonstrated a significant increase from 76 (±4) to 90 (±2) over a period of 6 months (see Table 2), and remained consistent at 12 months (95% CI, *p* < 0.001). This finding suggests an enhancement in the perception of respiratory symptoms and an improvement in quality of life.

### 3.7 Safety

AEs occurred in 14.8% (*n* = 4) of patients, all of which were reversible and mild to moderate in severity. The majority of patients who experienced AEs were effectively managed with dose modifications, which resulted in the resolution of symptoms. However, a skin rash was reported in a child following the administration of ETI. This patient discontinued treatment due to toxicity. He was treated with antihistamines and saline, and subsequently continued ETI with a reduced dosage. Two cases of elevated transaminase levels (AST/ALT: 100/110 U/L) were observed in adolescents after 3 and 6 months of treatment with ETI, respectively. When the dose was reduced by 50% and Ivacaftor administration was discontinued at night, transaminase levels decreased to within the normal range. Furthermore, one adolescent exhibited elevated serum creatine phosphokinase (CPK) levels of 550 U/L. Subsequent to a reduction in dosage, these levels returned to a value slightly above the normal range.

## 4 Discussion

This real‐world analysis highlights the clinical benefits of ETI therapy in pediatric PwCF, confirming findings from previous clinical trials while extending them to a heterogeneous, real‐life cohort [14, 15, 16, 17]. The observed improvement in ppFEV_1_ (mean absolute increase of 15% at 12 months) is consistent with prior controlled studies and emphasizes the rapid functional response across both age groups [5, 6]. These gains are consistent with the substantial improvements reported in recent real‐world evidence (RWE) cohorts from Europe and North America, which underscore the efficacy of ETI outside of trial settings [18]. This reinforces the hypothesis that clinical benefit from ETI is not strictly age‐dependent, although our dataset is limited to a narrow age range (6–17 years).

ETI therapy was well tolerated, overall safe with an acceptable side‐effect profile, and effective in improving lung parameters in our cohort of children and adolescents with CF.

All measured parameters exhibited notable enhancements in lung function, BMI, SwCl, PEx, and CFQ‐R scores within the initial six‐month period of therapy. Moreover, a positive trend was observed during the 12‐month follow‐up period following ETI treatment.

The overall increase in ppFEV_1_ in the entire study cohort after 24 weeks with ETI was 11%, with a further increase at 12 months (+15%). Additionally, ppFVC showed no significant changes (*p* = 0.30) and remained relatively stable over time, likely due to high baseline values.

Our results are comparable to those reported by Regard et al. and Olivier et al. [10, 11], who observed similar absolute gains in ppFEV_1_ within the first year of ETI treatment. The mean absolute change from baseline in lung function tests was not significantly different between our two age groups, supporting the hypothesis that the clinical benefit of ETI is independent of age. The pulmonary improvement observed in our group of PwCF aged 6–11 years was comparable to results from phase III clinical trials in pediatric patients carrying at least one Phe508del mutation [7, 8]. Our data from the 12–17‐year‐old group were consistent with pivotal trials in adolescents and adults [6].

A subgroup analysis stratified by lung disease severity revealed that subjects with ppFEV_1_ less than 70% and greater than 40% at baseline showed the most marked improvements, with significant absolute changes during the entire study period (+46%; *p* < 0.001). However, despite the results aligning with the hypotheses proposed by certain authors [11, 19], we suggest considering the possibility of bias due to the limited sample size of these subjects. Therefore, the results must be cautiously interpreted and confirmed on a larger population.

ETI demonstrated positive effects in reducing the number of pulmonary exacerbations (−27.6%), improving respiratory symptoms. This reduction aligns with real‐world evidence demonstrating that ETI therapy markedly decreases pulmonary exacerbation rates by approximately 40% in pediatric populations [20], with a similar magnitude observed across North American cohorts (rate ratio = 0.47) [21] as well as in adult populations with advanced lung disease [17]. These results suggest the efficacy of ETI in this subgroup of PwCF, in contrast to the moderate clinical improvements observed with other CFTR modulators [22, 23]. Similar results were recorded in a recent study including 34 children aged 6–11 years [24]. We agree with the authors that recurrent respiratory infections, which affect up to 25% of children in the first years of life, are generally self‐limiting and not CF‐dependent [20, 24]. Although PEx is an important indicator of disease severity in adults, it may be less reliable as a prognostic factor in the pediatric population.

The progressive increase in BMI suggests a potential improvement in growth parameters and nutritional status, independent of age group, which is supported by the gradual increase in weight *z*‐scores at 6 months and 12 months compared to baseline. Although these changes are positive, they were not statistically significant (*p* = 0.63). However, the height *z*‐score showed minimal changes from baseline, with slight improvements observed only in the 6–11 years cohort. Similar results are consistent with a phase III study [7]. It is important to consider individual variations in response to treatment, as children with CF often experience growth delays that may be more challenging to correct in the short term. Several studies have reported reduced circulating IGF‐1 levels in children with CF [25], suggesting that CFTR dysfunction may interfere with the GH–IGF‐1 axis. Evidence from animal models further indicates that loss of CFTR function in neurons can impair growth and contribute to endocrine dysregulation [26]. These observations point to a more complex pathophysiological mechanism that warrants further investigation.

The mean sweat chloride concentration in the overall population decreased significantly after 6 months (−44 mmol/L; *p* < 0.001), with values stabilizing at subsequent follow‐up, in line with clinical trials [6, 7, 8]. In both age groups, mean SwCl concentrations fell below the diagnostic threshold for CF by 6 months. This marked reduction suggests that ETI treatment corrects underlying CFTR dysfunction.

The rapid and sustained improvement in ppFEV_1_ was reflected in an increase in the CFQ‐R score, which increased by 14 points to a final score of 90 ± 2 (*p* < 0.001) at 12 months in the overall population. Both groups showed a significant change in the CFQ‐R score after starting ETI. These results highlight the key role of ETI in recovering respiratory function and improving quality of life.

No patients aged 6–17 years reported serious adverse events, demonstrating the good tolerability and safety profile of ETI in this cohort. Our findings reported in children were similar to that observed in a Phase III study [7]. A total of 14.8% of patients experienced adverse events, most of which resolved quickly with dose reduction. Toxicity was usually manageable through transient interruption or dose reduction. Notably, patients rechallenged at half‐dose after mild adverse events maintained clinically meaningful improvements at 12 months, indicating that dose adjustment may allow continued therapy without compromising efficacy. Elevation of liver enzymes and CPK are known side effects of CFTR modulators [6, 7, 8]; careful monitoring of ETI plasma concentrations is recommended to prevent similar episodes, especially in pediatric patients with a different pharmacokinetic profile [27]. Safety data remain immature, and longer follow‐up is needed to draw more definitive conclusions about the toxicity profile in a real‐world pediatric population.

It should be noted that this study is not without limitations. Potential limitations are the small sample size, the retrospective design and the lack of a control group, which limits the precision of the ETI treatment effect estimates. However, we attempted to overcome this bias by comparing two pediatric populations despite having different respiratory function characteristics at baseline.

A strength of our study is the focus on microbiological analysis of sputum before and after ETI treatment, stratified by age group and across the entire population. Significant reductions in *P. aeruginosa* and *S. aureus* positivity were observed in the overall setting after 12 months of ETI treatment, in agreement with some studies [28, 29] (Figure 1). The prevalence of common CF pathogens varied over time according to age (Figure 2). A higher colonization of *S. aureus* , *K. pneumoniae, rugose* and *mucoid P. aeruginosa* was observed in adolescents (12–17 years) compared with children aged 6–11 years. Overall, a reduction in all bacterial isolates was observed following ETI initiation (Figure 2). This age‐stratified pattern also extended to clinical outcomes: adolescents exhibited a higher baseline burden of PEx, consistent with their greater colonization rates, yet both age groups experienced a clear decline in exacerbations after ETI. Despite the modest absolute reduction in microbial colonization rates observed in the present cohort, even minor shifts in pathogen prevalence may hold clinical significance in pediatric CF setting. Persistent airway colonization, particularly with *S. aureus* and *P. aeruginosa* , is strongly associated with accelerated lung function decline, increased risk of PEx, and structural airway damage. Therefore, a measurable decrease in pathogen recovery following ETI may reflect an early biological signal of improved airway environment, potentially translating into long‐term clinical benefit if sustained. Importantly, these findings are aligned with larger real‐world studies reporting reduced infection‐related morbidity under ETI [17, 21, 28], reinforcing the clinical significance of incremental microbiological improvements in children and adolescents.

The discovery of triple therapy, combined with increasingly early neonatal screening, has resulted in a substantial enhancement in the survival rates of PwCF [30]. These improvements have a positive impact on both the symptomatological and psychological well‐being of pediatric patients, with improved quality of life and increased life expectancy. The reduction in PEx, resulting in fewer hospitalizations, enables children to attend school regularly and integrate fully into social life.

Furthermore, subjects with impaired lung function or at high risk of developing impairment showed a greater increase in ppFEV_1_ than patients with less critical clinical conditions at baseline [11, 19]. In this subset, early initiation of ETI may yield significant benefits in terms of rapid recovery of respiratory function and improvement in overall clinical status. It is conceivable that early use of ETI in subjects homozygous for the Phe508del CFTR, who are at high risk of severe manifestations, could prevent the onset of pancreatic insufficiency and potentially bronchiectasis, diabetes and other complications [31]. However, despite the therapeutic effects of ETI in slowing or, in some cases, halting disease progression, ETI cannot reverse established lung damage. Therefore, this subgroup should be evaluated carefully, aiming to start therapy before irreversible respiratory deterioration occurs. In newly diagnosed pediatric patients with acceptable clinical conditions, absence of bronchiectasis and microbial colonization, we recommend careful case‐by‐case evaluation before initiating ETI due to potential side effects mainly in the liver [6, 7, 8, 32].

Given the limited current evidence, further real‐world studies with larger datasets and longer follow‐up are required to validate these findings, considering the time of onset of any ETI‐related adverse event. Prospective studies incorporating pharmacogenomic assessments would help to clarify the impact of ETI on liver toxicity.

## 5 Conclusions

In this real‐world pediatric cohort, ETI therapy consistently improved lung function, quality of life, and microbiological outcomes, confirming its role as the standard of care for eligible PwCF. Sustained pathogen reduction and early clinical gains suggest potential long‐term benefits when treatment is initiated early. Despite its overall favorable safety and tolerability profile, elevations in liver enzymes warrant careful monitoring and pharmacokinetic‐guided dose optimization in pediatric patients.

## Author Contributions

N.P. wrote the manuscript; N.P., A.P., P.T., R.P., and G.C. designed the research; N.P., L.A.F., and G.C. performed the research; N.P., R.V., and R.G. analyzed the data.

## Ethics Statement

This study was reviewed and approved by the Ethics Committee of Lazio 1 (protocol number 7096/CE Lazio 1). All patients provided written informed consent to participate in the study and for their data to be published. All data collected were treated in accordance with current privacy regulations and Good Clinical Practice (GCP). Data were collected anonymously; each patient was assigned an identification code.

## Conflicts of Interest

The authors declare no conflicts of interest.

## Data Availability

The data that support the findings of this study are available on request from the corresponding author and are not publicly available due to privacy or ethical restrictions.
